# Synergistic structural and functional alterations in the medial prefrontal cortex of patients with high-grade gliomas infiltrating the thalamus and the basal ganglia

**DOI:** 10.3389/fnins.2023.1136534

**Published:** 2023-03-27

**Authors:** Zheng Yan, Jun Tang, Honglin Ge, Dongming Liu, Yong Liu, Hongyi Liu, Yuanjie Zou, Xinhua Hu, Kun Yang, Jiu Chen

**Affiliations:** ^1^Department of Neurosurgery, The Affiliated Brain Hospital of Nanjing Medical University, Nanjing, Jiangsu, China; ^2^Department of Neurosurgery, Yixing Hospital of Traditional Chinese Medicine, Yixing, China; ^3^Institute of Brain Functional Imaging, The Affiliated Brain Hospital of Nanjing Medical University, Nanjing, Jiangsu, China

**Keywords:** synergistic alteration, high-graded gliomas, thalamus, medial prefrontal cortex, DMN

## Abstract

**Background:**

High-grade gliomas (HGGs) are characterized by a high degree of tissue invasion and uncontrolled cell proliferation, inevitably damaging the thalamus and the basal ganglia. The thalamus exhibits a high level of structural and functional connectivity with the default mode network (DMN). The present study investigated the structural and functional compensation within the DMN in HGGs invading the thalamus along with the basal ganglia (HITBG).

**Methods:**

A total of 32 and 22 healthy controls were enrolled, and their demographics and neurocognition (digit span test, DST) were assessed. Of the 32 patients, 18 patients were involved only on the left side, while 15 of them were involved on the right side. This study assessed the amplitude of low-frequency fluctuation (ALFF), regional homogeneity (ReHo), gray matter (GM) volume, and functional connectivity (FC) within the DMN and compared these measures between patients with left and right HITBG and healthy controls (HCs).

**Result:**

The medial prefrontal cortex (mPFC) region existed in synchrony with the significant increase in ALFF and GM volume in patients with left and right HITBG compared with HCs. In addition, patients with left HITBG exhibited elevated ReHo and GM precuneus volumes, which did not overlap with the findings in patients with right HITBG. The patients with left and right HITBG showed decreased GM volume in the contralateral hippocampus without any functional variation. However, no significant difference in FC values was observed in the regions within the DMN. Additionally, the DST scores were significantly lower in patients with HITBG, but there was no significant correlation with functional or GM volume measurements.

**Conclusion:**

The observed pattern of synchrony between structure and function was present in the neuroplasticity of the mPFC and the precuneus. However, patients with HITBG may have a limited capacity to affect the connectivity within the regions of the DMN. Furthermore, the contralateral hippocampus in patients with HITBG exhibited atrophy. Thus, preventing damage to these regions may potentially delay the progression of neurological function impairment in patients with HGG.

## 1. Introduction

Gliomas are the most common primary intracranial tumors and account for 80.8% of malignant brain neoplasms (Ostrom et al., 2019). HGGs exhibit a rapid growth rate, leading to significant invasion and destruction of the brain tissue due to the quick expansion velocity. This leads to deteriorative neurological function in patients, such as noticeable cognitive deficits (Swanson et al., 2003; Omuro and De Angelis, 2013). Previous studies have demonstrated that the presence of the brain has an impact on lesions and the underlying processes. In response to the lesion, other brain regions may attempt to compensate through cortical reorganization or reassigning physiological resources (Fisicaro et al., 2016; Herbet et al., 2016). Neural adaptation involves the recruitment of four brain regions, which includes functional redistribution within and around the tumor as well as the recruitment of remote and contralateral areas within the lesioned hemisphere (Duffau, 2014; Liu D. et al., 2020). In addition, compensation of functional networks can occur within specific lesion regions (Zhang et al., 2018; Liu Y. et al., 2020). The response to the lesion suggests a universal protection phenomenon across different brain regions.

The basal ganglia, anatomically close to the thalamus, facilitate the execution of sensorimotor tasks combined with the thalamus. The thalamus, a core structure of the brain, modulates sensory message processing from different zones of the cerebral cortex and maintains conscious experience (Sherman and Guillery, 2002; Zhang et al., 2010). Thalamocortical systems and the DMN are associated with the state of consciousness (White and Alkire, 2003; Vanhaudenhuyse et al., 2010; Fernandez-Espejo et al., 2012). Although the thalamus is not generally considered part of the DMN, there are high levels of structural and functional connectivity between the thalamus and the central regions of the DMN. These regions include the angular gyrus (AG), hippocampus, precuneus, midcingulate cortex (MCC), medial prefrontal cortex (mPFC), and superior frontal gyrus (sFG) (Cunningham et al., 2017). The DMN plays a central role in higher cognitive processing, as evidenced by its anatomical position and previous research findings. Furthermore, it has been associated with many diseases (Anticevic et al., 2012; Vatansever et al., 2015). Gliomas that invade the basal ganglia inevitably infiltrate the thalamic region, leading to abnormal brain activity due to the high degree of tissue invasion by HGG (Huse et al., 2011). Extensive edema surrounding HGGs usually contains tumor cells that create a suitable niche and provide them with the necessary nutrition (Engelhorn et al., 2009; Lin, 2013). Thus, there is a great need to assess potential functional and structural changes at the whole brain level since the tumor can invade not only local regions but also the peripheral and distant regions. Furthermore, when confronted with brain neoplasms, it has been observed that large-scale neural networks, such as the DMN, the language network, and the cognitive control network, undergo structural and functional alterations and reorganization (Esposito et al., 2012; Zhang et al., 2018; Liu Y. et al., 2020). These findings suggest that the neural networks may play a crucial role in maintaining the robustness of the central nervous system. However, whether the main regions of the DMN support the structural and functional reorganization in patients with HITBG is not clear.

In recent years, there has been a growing use of resting-state functional magnetic resonance imaging (rs-fMRI) to study abnormal brain function. This technique allows researchers to track local alterations in blood oxygenation, which correlate with changes in brain activity, in a rapid and non-invasive manner (Matthews et al., 2006). The amplitude of low-frequency fluctuation (ALFF) and the regional homogeneity (ReHo) of the time series have been used as metrics to assess spontaneous brain activity (Cordes et al., 2001; Zang et al., 2004; Yang et al., 2007). Furthermore, structural MRI has been used to measure gray matter volume to investigate structural plasticity in patients with cerebral gliomas. These indicators, each with different advantages, can be used to better assess patients' functional and structural changes on a global scale.

In this study, we focused on exploring functional compensation in patients with HITBG by measuring ALFF and ReHo within the DMN. Additionally, we conducted FC analysis to assess the relationship between different regions within the DMN while also using GM volume to investigate the potential association between structural plasticity and function. Neurocognitive data, such as the digit span test, was also used to investigate the relationship between cognitive ability in the patients and HCs. This study can provide new insights into the impact of neural networks on the brain when significant local regions are infiltrated by highly invasive gliomas, shedding light on the relationship between the DMN and HGG. Moreover, this study can serve as an important guide in the treatment of patients with HGG, depending on the specific brain regions that play a vital role in the compensatory response to injury.

## 2. Materials and methods

### 2.1. Subjects from the Nanjing brain hospital-brain tumor neuroimaging project (NBH-BTnp)

The present study enrolled 33 patients [age (mean ± SD) 57.18 ± 11.32 years, 23 men and 10 women] with highly invasive HGGs affecting the thalamus (WHO III/IV). These patients underwent therapy at the Department of Neurosurgery, the Affiliated Brain Hospital of Nanjing Medical University. Of the 33 patients included in the study, 18 [age (mean ± SD) 60.39 ± 8.98 years; 14 men and four women] of them with left and 15 [age (mean ± SD) 53.3 ± 12.88 years; nine men and six women] of them with right glial tumors met the following criteria: (1) patients with histopathologically confirmed high-grade primary gliomas (based on the 2016 WHO classification system) (Louis et al., 2016); (2) patients with gliomas affecting the unilateral thalamus and including perifocal edema; (3) patients with no significant history of craniocerebral injury, cerebrovascular disease, stroke, or any neurologic or psychiatric disease; and (4) patients with no history of brain radiotherapy or chemotherapy. Moreover, the subjects with no evident midline shift (<10 mm, septum pellucidum, corpus callosum, third ventricle) were included, considering the thalamus is located near the midline of the cerebrum. The exclusion criteria were as follows: (1) patients with a recurrent glial brain tumor; (2) patients with multiple lesions; and (3) patients with deficient MRI examinations or preprocessing. In addition, this study recruited 24 healthy controls [age (mean ± SD) 56.27 ± 6.36 years, with 12 men and 10 women] who were matched for age and gender with the patients. They were recruited from the local community and had no history of any neurologic or psychiatric illnesses. All the subjects were right-handed based on the handedness scale.

The Ethics Committee of the Affiliated Brain Hospital of Nanjing Medical University approved this study. Written informed consent was obtained from all the subjects.

### 2.2. MRI data acquisition

MRI data were obtained from preoperative scans of patients between 2013 and 2022. The 3.0 T Verio scanner was used to obtain scans from the Affiliated Brain Hospital of Nanjing Medical University.

Structural images of the entire brain were acquired using the three-dimensional (3D) T1-weighted sequence. The detailed scan parameters were as follows: voxel size = 1 × 1 × 1mm^3^, repeat time = 1.900 ms, echo time = 2.49 ms, inversion time = 900 ms, acquisition matrix = 256 × 256, flip angle = 9°, slice thickness/gap = 1/0.5 mm, and slice number = 176. Additionally, conventional T2-weighted (T2W) images were collected for reference.

Blood Oxygen Level Dependent (BOLD) signals of rs-fMRI were measured using an echo-planar image sequence with two sets of functional scan parameters, such as the repetition times = 2,000/2,000 ms, echo times = 30/30 ms, flip angles = 90°/90°, the acquisition matrices = 64 × 64/64 × 64, resolution = 3.75 × 3.75 × 4 mm/3.4 × 3.4 × 4 mm, fields of view (FOV) = 240 × 240 mm/220 × 220 mm, the number of time points = 140/240, slice numbers = 30/36, slice thicknesses = 3.0/4.0 mm, and slice gaps = 4/0 mm. Among the sets, the first was scanned between 2013 and 2016, and the second was scanned between 2017 and 2022. The second set obtained rs-fMRI data from the HC subjects. Over time, our research team optimized and improved different parameters in the imaging protocol. In addition, we included homogeneous parameter differences in the same scanner as a covariate in the general linear model (GLM) to account for potential confounding factors.

### 2.3. Tumor drawing

**Figure 1:**
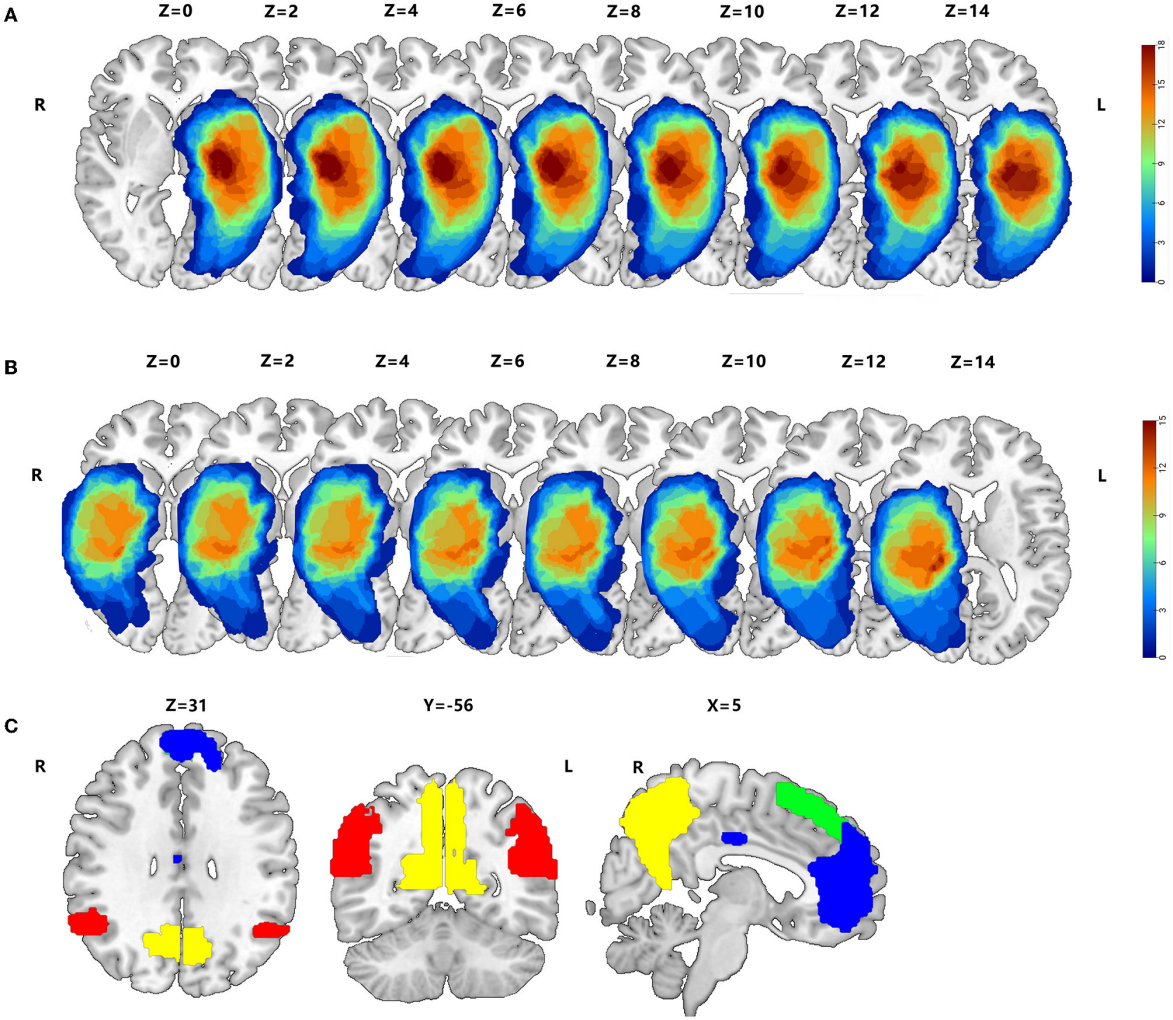
Overlap maps of HGGs, such as peritumoral edema, between the left hemisphere **(A)** and right hemisphere **(B)**. **(C)** The AG, hippocampus, precuneus, mPFC, MCC, and sFG within the DMN are based on the Harvard–Oxford Cortical of FSL, whose threshold is 25% signal intensity, and the Stanford FIND Lab functional ROI database.

3D T1 enhancement images for each patient were registered to the Montreal Neurological Institute (MNI) template using the standard non-linear spatial normalization algorithm, which was provided by SPM12 to define the lesion site of the brain in patients with HGG. Due to the characteristics of significant destruction and widespread infiltration into the surrounding tissue, the perifocal edema around the tumor, which inevitably injures the function of lesions, was included in the tumor masks. The lesion outline was traced manually on individual 3D T1-weighted images combined with T2 images using the Itk-Snap software. All tumor masks were overlapped in the Ch2better template using the MRIcroGL software (Figures 1A, B).

### 2.4. Selection of regions of interest within the DMN

To explore functional compensation in patients with HITBG, we obtained six standard masks within the DMN using the Harvard–Oxford Cortical of FSL, based on previously published studies (Cunningham et al., 2017). These masks were obtained with a threshold of 25% signal intensity, and we also used the Stanford FIND Lab functional region-of-interest (ROI) database (http://findlab.stanford.edu/functional_ROIs.html). Furthermore, all ROIs, including the AG, the hippocampus, the precuneus, the mPFC, the MCC, and the sFG, were superimposed on a T1 2 mm resolution MNI152 of a standard template brain (Figure 1C). Moreover, unilateral injury masks were removed depending on the nidus in the left and right cerebrum, respectively. Consequently, the right AG (rAG), the right hippocampus, the right precuneus, the mPFC, the MCC, and the sFG were defined as the mask of left gliomas. In contrast, the left AG (lAG), the left hippocampus, the left precuneus, the mPFC, the MCC, and the sFG were selected as the masks of right gliomas.

### 2.5. Neurocognitive test

The DST (which includes the DS forward and backward) was used as a neuropsychological tool to assess short-term verbal memory (Richardson, 2007). All the HC subjects and some patients underwent the DST (Table 1).

**Table 1:** Demographic characteristics of HGGs and healthy controls (HCs).

**items**	**HCs**	**ALL HGGs**	**Left HGGs**	**Right HGGs**	*p*-Value^*^	***p*-Value^†^**	***p*-Value^‡^**
Total, n	22	33	18	15	NA	NA	NA
Age (Mean ± SD), years	56.27 ± 6.36	57.18 ± 11.32	60.39 ± 8.98	53.3 ± 12.88	0.73	0.10	0.36
Gender (M/F), n	12/10	23/10	14/4	9/6	0.25	0.13	0.74
Education levels (Mean ± SD), years	12.66 ± 2.64	7.76 ± 4.10	8.33 ± 3.65	7.07 ± 4.62	<0.01	<0.01	<0.01
MRI parameters (type1/type2), n	22/0	22/11	12/6	10/5	NA	NA	NA
TBV (Mean ± SD),cm^3^	1,399.79 ± 103.87	1,369.67 ± 135.31	1,414.32 ± 112.04	1,316.09144.81	0.36	0.67	0.07
DST (Mean ± SD), score	12.68 ± 2.46	9.11 ± 3.18	NA	NA	0.01	NA	NA

All the *p*-values were obtained with the *t*-test except for gender (chi-square test). All HCs and nine HGGs completed the digit span test. Thus, a different analysis of neurocognitive data was applied to all the patients with HGGs and HCs.

* Difference analysis between all the patients with HGGs and HCs.

† Difference analysis between the patients with left HGGs and HCs.

‡ Difference analysis between the patients with right HGGs and HCs.

### 2.6. MRI imaging data preprocessing

We used a reliable tool, Data Processing and Analysis for Brain Imaging (DPABI; http://www.rfmri.org) (Yan et al., 2016) to preprocess each scan, depending on the Statistical Parametric Mapping 12 (SPM12; http://www.fil.ion.ucl.ac.uk/spm) toolkit within MATLAB2014a (http://www.mathworks.com/products/matlab/).

In structural image preprocessing, the images were manually adjusted and shifted to the anterior commissure, which was defined as the origin (mm coordinates: 0, 0, 0). We partitioned the structural images into GM, white matter (WM), and cerebrospinal fluid (CSF). Then, they were spatially normalized to MNI-152 standard space with an isotropic voxel resolution of 1.5 × 1.5 × 1.5 mm using linear affine registration and non-linear deformation. The normalized data were modulated in this course to preserve the original voxel information. In addition, the Diffeomorphic Anatomical Registration Through Exponentiated Lie Algebra (DARTEL) algorithm was used in image registration to reduce deformations. Finally, the GM maps from all the subjects were smoothed using a 6-mm fullwidth at half maximum (FWHM) Gaussian kernel.

The first 10 functional volumes were removed for all subjects to account for the initial magnetization equilibrium and eliminate any potential mechanical noise. We used several methods, including slice timing and realignment, to correct for temporal differences and head motion. In addition, subjects with a head motion exceeding 3 mm in translation or 3° in rotation were excluded from the study. The remaining functional images were manually reoriented and shifted as needed. To standardize the images, the functional images were normalized to the MNI space using the normalization parameters determined by the DARTEL algorithm. The process was completed after the structural image was coregistered with the mean functional image and segmented. The normalized images were resampled using an isotropic voxel resolution of 3 × 3 × 3 mm. Nuisance covariates, including Friston's 24-head motion parameter, WM, and CSF, were regressed out. Spatial smoothing with a 6-mm FWHM was applied before measuring the ALFF and seed-based functional connectivity of all scans and after calculating ReHo. Finally, low-frequency band screening (0.01–0.1 Hz) was applied to eliminate physiological and scanner noise at low and high frequencies.

### 2.7. Neural activity analysis of DMN regions without lesions

The time courses for each voxel were switched to the frequency domain with a fast Fourier transform algorithm. Each power spectrum frequency was proportional to the square of the corresponding amplitude. The amplitude of each voxel at 0.01–0.1Hz was determined and considered the ALFF of that voxel. Additionally, the ReHo within the time series in rs-fMRI between each voxel and its neighbors was measured using Kendall's coefficient concordance (KCC). It was standardized by dividing the KCC of each voxel by the average KCC for the entire brain.

### 2.8. ROIs-based DMN regions FC analysis

Based on the differences in ALFF and ReHo within the DMN between patient groups, we defined ROIs that depicted the overlap of functional abnormality between the left and right HITBG. Individual averaged time series of these selected ROIs were extracted as a reference to perform Pearson's correlation analysis on the averaged time courses of other seeds. Finally, Fisher's z-transformation helped improve the normality of the correlation coefficients for additional *t*-tests.

### 2.9. VBM analysis

The methods of voxel-based morphometry (VBM) were extensively used to compare the concentration of the gray matter between groups across various diseases (Ashburner and Friston, 2000; Du et al., 2014; Hu et al., 2020). We measured GM volume within the DMN in each subject and compared the difference between patients and HCs to explore the potential structural effect. In addition, the summation of GM, WM, and CSF volumes was considered the total brain volume (TBV).

### 2.10. Statistical analysis

The study compared demographic variables and DST scores between patients (left and right HITBG) and HCs using IBM SPSS software. An independent two-sample *t*-test was used to analyze the age, gender, and DST score differences between the groups. In contrast, the gender variable was compared using the chi-square test. A *P*-value of more than 0.05 was used as the statistical significance threshold. The correlation between DST scores and functional and structural indexes in patients was analyzed.

In addition, the ALFF, ReHo, GM, and FC values of each voxel among the patients (left and right HITBG) and HCs were transformed into z-values. Then, they were compared using two-sample *t*-tests with covariates such as age, gender, education (years), and two types of parameters of the MRI protocol (TBV was considered a covariate in VBM analysis) depending on the DMN regions without lesions through the DPABI toolkit. The threshold-free cluster enhancement family-wise error (TFCE-FWE) correction rectified the results. The number of permutation tests was set at 5,000. The statistical significance of the cluster results was determined by correcting the *p*-values using the TFCE-FWE method, and only those clusters with a corrected *p*-value of less than 0.05 were considered statistically significant.

## 3. Results

### 3.1. Demographic and cognitive characteristics

The characteristics of all the subjects are exhibited in Table 1. No significant differences in age or gender were observed between the patients (left and right HITBG) and HCs. The education level of HCs was higher than that of patients (*p* < 0.05). Compared to the HCs, the scores were significantly lower in patients with HITBGs (*p* < 0.05).

### 3.2. Alteration of the GM volume

**Figure 2:**
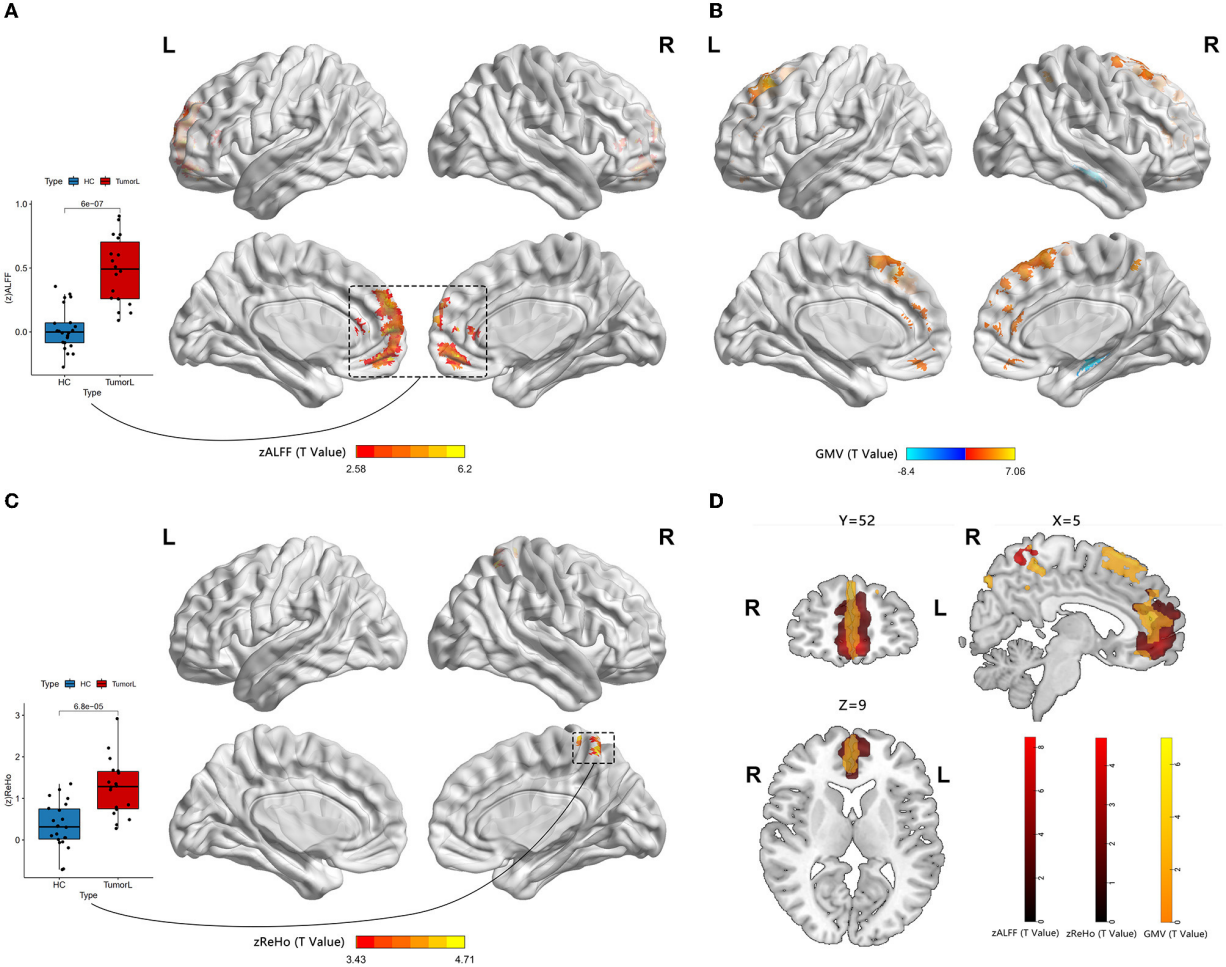
Neural activity and VBM analysis between HGGs in the left hemisphere and HCs. **(A, C)** Red indicates elevated ALFF and ReHo within the DMN (permutation test, TFCE-FWE corrected a *p*-value of < 0.05). The boxplot reveals differences within the means zALFF and zReHo extracted from the red cluster. **(B)** Red and blue represent elevated and reduced GM volume, respectively. **(D)** An overlap map among the ALFF, ReHo, and GM volume.

In the right HITBG (*n* = 15), the map of the individual lesion overlap was displayed (Figure 1A). Compared with the HCs group, HGG patients showed a higher GM volume in the mPFC and sFG within the DMN. However, GM volume was found to be decreased in the contralateral hippocampus when tumors invaded the right thalamus and the basal ganglia (Figure 2B).

**Figure 3:**
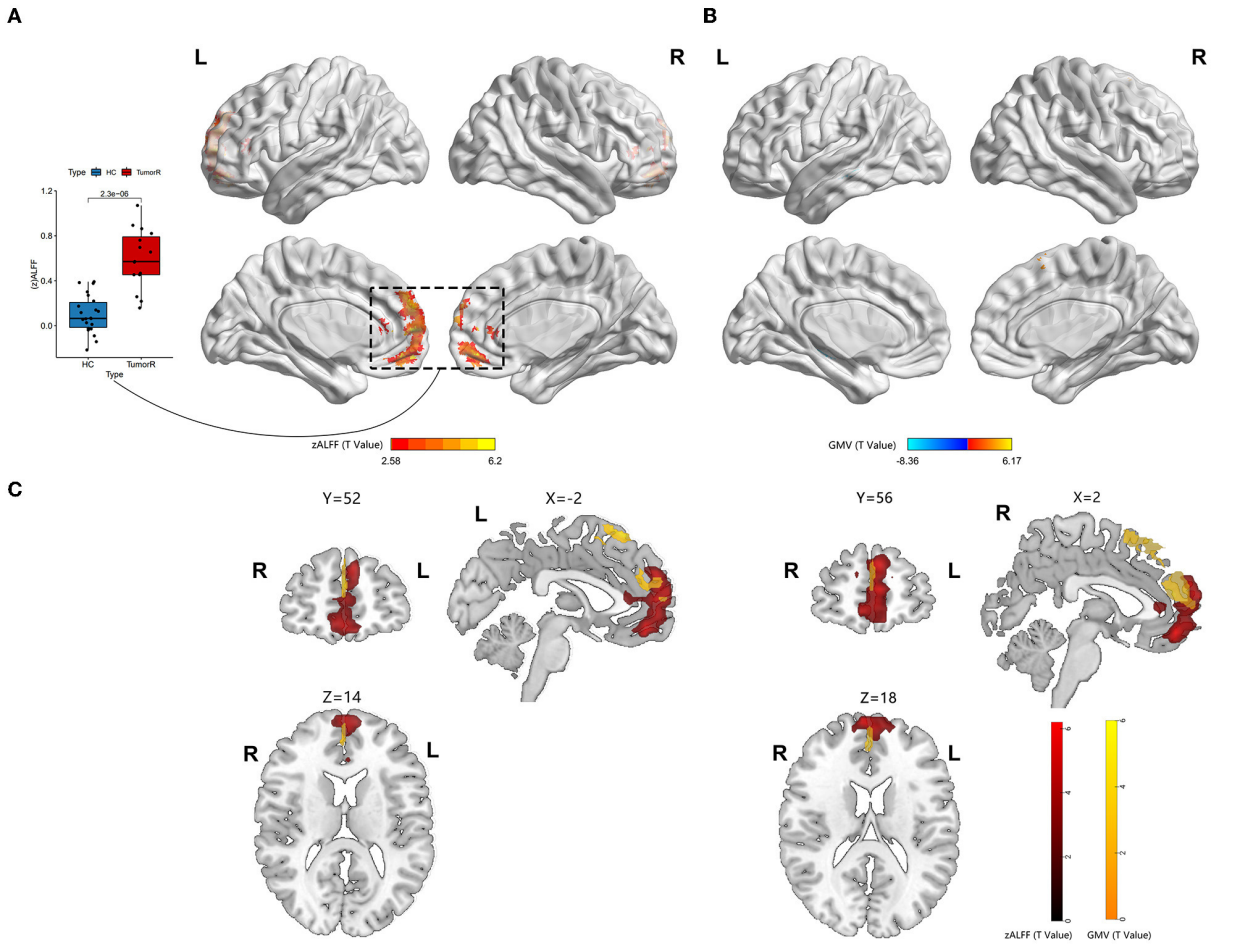
Neural activity and VBM analysis between HGGs within the right hemisphere and HCs. **(A)** Red indicates elevated ALFF within the DMN (permutation test, TFCE-FWE corrected *p* < 0.05). The boxplot shows differences in the mean zALFF extracted from the red cluster. **(B)** The red and blue bars indicate increased and decreased GM volume, respectively. **(C)** Overlap map between the ALFF and GM volume.

Furthermore, the map of individual lesion overlap within the left HITBG (*n* = 18) is shown in Figure 1B. The results indicated that patients with left thalamus and basal ganglia injuries had significantly increased GM volume in the mPFC, sFG, and contralateral precuneus within the DMN. However, a decreased GM volume was observed in the contralateral hippocampus (Figure 3B, Table 2).

**Table 2:** The differences between HGGs and HCs through neural activity and VBM analysis.

**Group**	**Variation analysis**	**Regions**	**Cluster size (voxels)**	**Peak MNI coordinate**			***T*-Value**
				**X**	**Y**	**Z**	
HCs vs LHITBG	ALFF	Medial Frontal Gyrus	533	−3	51	9	8.47
		Superior Frontal Gyrus	68				
	ReHo	Precuneus_R (aal)	26	6	48	69	4.71
	GM volume	Medial Frontal Gyrus	1968	28.5	13.5	64.5	7.06
		Superior Frontal Gyrus	2247	1.5	−37.5	54	6.67
		Precuneus_R (aal)	559				
		Hippocampus_R (aal)	248	25.5	−15	−13.5	−8.40
HCs vs RHITBG	ALFF	Medial Frontal Gyrus	295	0	48	−12	6.20
	GM volume	Superior Frontal Gyrus	86	0	57	22.5	4.87
		Frontal_Sup_Medial_L (aal)	414				
		Superior Frontal Gyrus	284	1.5	30	60	6.17
		Hippocampus_L (aal)	70	−34.5	−30	−7.5	−8.36

LHITBG, HITBG in the left hemisphere; RHITBG, HITBG in the right hemisphere.

### 3.3. Group differences between ALFF and ReHo value

A significantly increased ALFF value in the mPFC (containing a small overlap area of sFG) within the DMN (permutation test, TFCE-FWE corrected, *p* < 0.05) (Figures 2A, 3A, Table 2) was found in patients with left and right HITBG compared with HCs. Furthermore, the significantly elevated ReHo value in the contralateral precuneus within the DMN was observed only in patients with the left HITBG (permutation test, TFCE-FWE corrected, *p* < 0.05) (Figure 2C, Table 2). In contrast, no significant difference in ReHo value was observed in the regions within the DMN between patients through the right HITBG and HCs.

### 3.4. Variation of functional connectivity

The patients with left and right HITBG exhibited a significant variation index in functional activity compared to the HCs selected as the ROI. Specifically, the ALFF map in the mPFC within the DMN was elevated. However, no significant difference in the FC value was found within the DMN regions.

### 3.5. Association among DST and MRI indexes

No significant correlation was observed between the DST score and the ALFF value. The ALFF value was extracted based on the abnormal brain activity observed in both the left and right HITBG. Similarly, no significant association was found between DST and the GM volume measured based on the shared abnormal structure in the left and right HITBG.

## 4. Discussion

Brain plasticity is a compensatory mechanism that responds to injury and optimizes neural function. When injuries are chronic, such as low-grade tumors with slow growth, effective recruitment and functional reorganization occur in the other brain regions (Kong et al., 2016; Zhang et al., 2018). However, little is known about the potential for plasticity during rapid brain injury. This study is the first to investigate whether the DMN has functional and structural reorganization potential in HITBG patients. We found significantly elevated ALFF clusters in the mPFC within the DMN when we performed the variation analysis among patients with the left and right HITBG and HCs, respectively. Moreover, we found a significant increase in the ReHo value in the contralateral precuneus within the DMN only in patients with left HGG. However, no significant alteration in the FC value was observed in the DMN regions with mPFC as a mask in HGG. Interestingly, the overlap maps indicated a similar pattern of increased GM volume with neural activity in mPFC, suggesting the possible critical role of mPFC in reorganization.

The consistent result of significantly elevated ALFF in the left and right HITBG indicates that the mPFC, as a remote region, is vital in compensating for the functional deficit resulting from injury to the thalamus and the basal ganglia. Existing research has shown that the complex brain circuit among the cortex, basal ganglia, and thalamus is critical in processing sensorimotor tasks (Smith et al., 2011; Riva et al., 2018). The lesions in the basal ganglia and the thalamus can lead to neuropsychological and consciousness deficits (White and Alkire, 2003; Riva et al., 2018). However, due to the rapid and uncontrolled nature of brain injury in HGG patients, their neurological deficits may outweigh the potential for plasticity (Kong et al., 2016). This finding is consistent with our result that executive functions decline in patients with HGG. In addition, the mPFC plays a critical role in decision-making and memory, similar to the function deficit after basal ganglia and thalamus injuries (Euston et al., 2012). The thalamus can also show abnormal feedback when there is damage to the PFC (Kim et al., 2011). Thus, structural and functional interactions among the cortex, basal ganglia, and thalamus could provide the potential foundation for reorganization. This study demonstrated the presence of remote plasticity within the DMN regions when the core region was impacted by HGG invasion. However, the right precuneus in patients with left HITBG showed more synchronized spontaneous activity. It has been speculated that the diversity of individual injury and high heterogeneity of HGGs cause a difference in results (Huse et al., 2011; Kristensen et al., 2019). In addition, the increased homogeneity in the precuneus provides complementary evidence for revealing alterations in spontaneous brain activity. Previous research on the structural and functional connectivity between the core of the DMN, including the mPFC and precuneus, and the thalamus suggests a potential neural mechanism (Cunningham et al., 2017).

In addition, our research demonstrated a structural alteration within the DMN after HITBG. This result indicated that the measurement of function and structure increased in similar and close regions, which was consistent with the alteration reported in a previous study (Zhang et al., 2018). This may suggest that there was a reorganization of neural activity that is synchronized with the structural alteration. Therefore, the overlap between the increased structure and function, including in the mPFC and precuneus, could be an active participant in the process of HITBG-induced plasticity.

In this study, the patients with left and right HITBG had decreased GM volume in the contralateral hippocampus. However, there was no change in function or activity within the hippocampus. This result suggests that HGGs infiltrating the unilateral basal ganglia, thalamus, and surroundings may lead to contralateral hippocampus structural atrophy within the DMN instead of neuroplasticity. Given the uncontrolled proliferation and rapid invasion of HGGs, microenvironment alteration, a lack of plasticity, and inflammation could be potential factors contributing to atrophy (Engelhorn et al., 2009; Liu et al., 2012; Martin-Noguerol et al., 2021). Previous research has shown a similar result in the hippocampus when the medial temporal lobe, anatomically close to the thalamus and basal ganglia, is invaded by HGGs (Yuan et al., 2020). The hippocampus supports the mPFC in the mnemonic functions of the brain (Euston et al., 2012). However, no FC alteration was found between these regions.

In brief, the most notable finding of this study was the combined increase in both structural and functional activities in the mPFC of patients with unilateral HITBG. The mPFC, the central hub of the DMN, is characterized by the complex functions of memory retrieval, social cognition, and affective processing (Lieberman et al., 2019; Muller et al., 2020). The mPFC exhibited a considerable potential for synergistic plasticity in response to rapid and extensive lesions. In addition, the synergistic effect between structure and function observed in this study is consistent with findings from other neuroimaging studies (Zhang et al., 2018; Li et al., 2020; Liu Y. et al., 2020) and may be an inherent mechanism to help prevent brain damage. This may explain why patients with HGG still retain some cognitive ability. However, the exact mechanism underlying this synergistic effect requires further research.

In this study, certain limitations should be emphasized. First, we used two types of resting-state functional MRI protocols in the MRI data of HGG patients, which were optimized. The two types of protocol were taken into account as a covariate of non-interest in the GLM analysis. Second, the education levels of the patients and the HCs did not match and were considered a covariate. However, even though the education levels of patients were lower than those of HCs, the functional activity and the GM volume of essential regions in patients still increased compared to HCs with a high education level. Finally, the sample size of the study needs to be increased to allow for more detailed grouping, and further research is required to fully understand the phenomenon of neuroplasticity.

## 5. Conclusion

The current study found that, when HITBG occurred, structural and functional alterations within the DMN were observed.

The ALFF and GM volume showed an increase in the mPFC of both left and right HITBG with significant overlap. Additionally, increased ReHo with matched GM volume was only observed in the left HITBG. Decreased GM volume was observed in the contralateral hippocampus, but no FC alteration was detected, indicating that HITBG has a limited effect on the association between brain regions. These findings may provide novel insights into the synchrony pattern between structure and function in neuroplasticity. Therefore, avoiding injury to these regions could potentially delay the development of neurological function damage in HGG patients.

## Data availability statement

The raw data supporting the conclusions of this article will be made available by the authors, without undue reservation.

## Ethics statement

This study was approved by Ethics Committee of the Affiliated Brain Hospital of Nanjing Medical University and Written informed consent from all participants was obtained.

## Author contributions

ZY, JT, and HG collected and analyzed the MRI data. DL, KY, JC, and YL conceived and designed the experiments. HL, YZ, and XH contributed to the analysis tools. ZY, HG, DL, KY, JC, and YL prepared the article, figures, and tables. All authors contributed to the article and approved the final version of the manuscript.
